# Innovative multidimensional models in a high-throughput-format for different cell types of endocrine origin

**DOI:** 10.1038/s41419-022-05096-x

**Published:** 2022-07-25

**Authors:** Stefan Bornstein, Igor Shapiro, Maria Malyukov, Richard Züllig, Edlira Luca, Evgeny Gelfgat, Felix Beuschlein, Svenja Nölting, Alfredo Berruti, Sandra Sigala, Mirko Peitzsch, Charlotte Steenblock, Barbara Ludwig, Patrick Kugelmeier, Constanze Hantel

**Affiliations:** 1 Department of Endocrinology, Diabetology and Clinical Nutrition, University Hospital Zurich (USZ) and University of Zurich (UZH), Zurich, Switzerland; 2 Medizinische Klinik und Poliklinik III, University Hospital Carl Gustav Carus Dresden, Dresden, Germany; 3 Endocrine Research Unit, Medizinische Klinik und Poliklinik IV, Klinikum der Universität München, 80336 Munich, Germany; 4 Department of Medicine IV, University Hospital, LMU Munich, Ziemssenstraße 1, 80336 München, Germany; 5 Oncology Unit, Department of Medical and Surgical Specialties, Radiological Sciences, and Public Health, University of Brescia at ASST Spedali Civili di Brescia, 25123 Brescia, Italy; 6 Section of Pharmacology, Department of Molecular and Translational Medicine, University of Brescia, 25123 Brescia, Italy; 7 Institute of Clinical Chemistry and Laboratory Medicine, University Hospital Carl Gustav Carus at Technische Universität Dresden, Dresden, Germany; 8 Kugelmeiers AG, Erlenbach, Switzerland

**Keywords:** Experimental models of disease, Translational research

## Abstract

The adrenal gland provides an important function by integrating neuronal, immune, vascular, metabolic and endocrine signals under a common organ capsule. It is the central organ of the stress response system and has been implicated in numerous stress-related disorders. While for other diseases, regeneration of healthy organ tissue has been aimed at such approaches are lacking for endocrine diseases - with the exception of type-I-diabetes. Moreover, adrenal tumor formation is very common, however, appropriate high-throughput applications reflecting the high heterogeneity and furthermore relevant 3D-structures in vitro are still widely lacking. Recently, we have initiated the development of standardized multidimensional models of a variety of endocrine cell/tissue sources in a new multiwell-format. Firstly, we confirmed common applicability for pancreatic pseudo-islets. Next, we translated applicability for spheroid establishment to adrenocortical cell lines as well as patient material to establish spheroids from malignant, but also benign adrenal tumors. We aimed furthermore at the development of bovine derived healthy adrenal organoids and were able to establish steroidogenic active organoids containing both, cells of cortical and medullary origin. Overall, we hope to open new avenues for basic research, endocrine cancer and adrenal tissue-replacement-therapies as we demonstrate potential for innovative mechanistic insights and personalized medicine in endocrine (tumor)-biology.

## Introduction

Spheroids and organoids have become increasingly popular to study tumor development and organogenesis and have opened avenues to drug discovery and personalized medicine. In cancer biology, multiple studies have demonstrated the genetic and histological comparability between cultured tumor spheroids and the original tumor [1–5]. As such, 3D cultures are being used to better understand the response to various drugs and therapeutics, involving various additional factors such as tumor microenvironment and multi-layer structures, thereby, leading in best case to individualized treatments for solid tumors [6].

However, the future of these multifaceted structures in the endocrine field goes far beyond and includes transplantation and organ replacement in humans in certain pathologies, such as type I diabetes, adrenal insufficiency or refractory Cushing’s disease [7–9]. Moreover, in islet research, which lacks different suitable cell lines, intact islets can be isolated from multiple donor specimens and maintained in vitro. However, these are impermeable to genetic manipulations since viral transduction only reaches the outermost cells, while the inner regions of the islets are not affected [10]. Dispersion of the islets and their subsequent re-aggregation into spheroids allows for the manipulation of all cells and the consecutive study within a multi-layer structure.

Similarly, in vitro models of adrenal origin have also suffered from a lack of suitable human cell lines that resemble functional properties of benign and malignant tissue origin for many years. Fortunately, this gap could recently been narrowed with cell models that are now applicable for a spectrum of research applications related to adrenocortical carcinoma [11–21]. However, there is still potential for improvement by translating adrenocortical cells towards 3D culture and appropriate personalized approaches in vitro. Moreover, for the diverse types of adrenocortical adenoma and paraganglioma/pheochromocytoma, appropriate in vitro models of human origin are still urgently needed [22].

Overall, there are manifold preclinical and clinical implications for tumor spheroid and organoids derived from human endocrine tissue origins. Thus, innovative high throughput applications for 3D models of endocrine origin open new avenues for endocrine cell replacement therapies and other personalized therapeutic approaches.

## Results

### Plate design

**Fig. 1:**
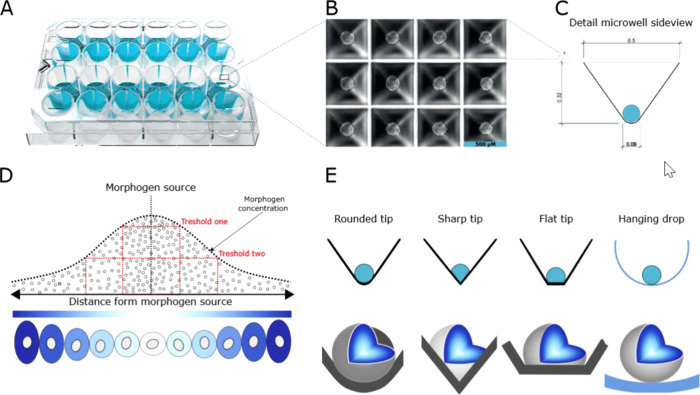
Plate design, geometry and resulting scientific advantages. Layout of the Sphericalplate 5D highlighting the 12 working wells **A**. Image of spheroids formed in the microwells within one of the wells of the plate **B**. Shape and measurements of the microwell **C**. Schematic illustration of a concentration gradient of morphogen **D** [modified from 23]). Advantage of rounded-tip design of microwell on spheroid formation **E**.

The Sphericalplate 5D is a 24-well laboratory plate with specially designed and patented microwells (Fig. 1A).

Each well is set up to allow the simplified establishment of 750 uniform and size-controlled pseuo-islets or tumor spheroids with only one pipet hub of a respective cell suspension (Fig. 1B). The spherical geometry with square top openings ensures that all cells participate in spheroid formation (Fig. 1C). The top square openings then merge into rounded tips to ensure the creation of uniform spheroids with cellular functionality and viability. This is achieved serving general biological principles like free energy minimisation with optimal tip geometry and special wall angles supporting spheroids but not limiting them. In addition, a patented medical grade ultra-low attachment coating ensures minimal surface interaction. Accordingly, compared to other available plate types and for any type of 3D-application, there is no need for anti-adherence rinsing solutions that could interfere with cell biology or hamper medical use. It is known, that the concentration of signalling molecules decides the cell fate (schematical representation Fig. 1D modified by [23]. Sharp or flat tips in classical microwell platforms interfere with this process and do not allow equal fate decisions because of unequal signalling molecule concentrations (Fig. 1E). Thus, the tip geometry and coating of the Sphericalplate 5D was specifically designed to support this process.

### Establishment and characterization of pancreatic pseudoislets

**Fig. 2:**
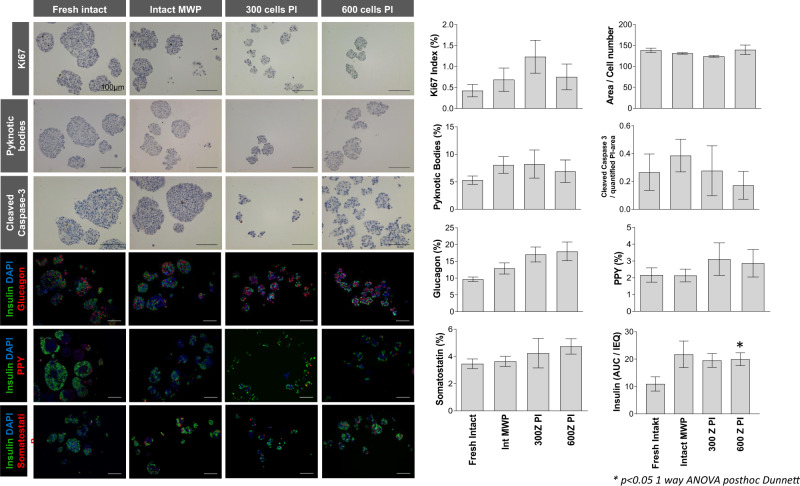
Freshly isolated islets were either cultured intact (fresh islets), in multiwell plates (Intact MWP) or dissociated to form pseudoislets (PI) constituting of 300 cells (300 cells PI) or 600 cells (600 cells PI). Islets or pseudoislets from each condition were processed for IHC for various markers, scale bar 100 um. Cells positive for the markers were counted and reported as percentage of all cells. Data obtained from 10 individuals (*n* = 10; including furthermore quantifications from three high power fields per individual) is reported as mean +/− sem and was analyzed by ANOVA with Dunnett test *p < 0.05.

In a first step, human pancreatic islets were plated either in commonly available cell culture dishes (fresh intact), multiwell plates (Intact MWP) or as re-aggregated pseudoislets on Sphericalplates (300 or 600 cells per pseudoislet; Fig. 2). Afterwards, important basal characteristics such as Ki67 as proliferation marker, the islet area per cell number, pyknotic bodies and Cleaved-Caspase-3 as markers reflecting cell death, furthermore Glucagon, PPY, Somatostatin and Insulin were determined. Subsequent quantifications revealed overall constant parameters with the exception of basally increased insulin levels for pseudoislets consisting of 600 cells each (Fig. 2). Thus, our experiments confirmed general applicability for high throughput establishment of pseudo-islets in round tip wells without any need for additional anti-adherence buffers.

### Establishment and characterization of adrenal tumor spheroids from adrenocortical cell lines

**Fig. 3:**
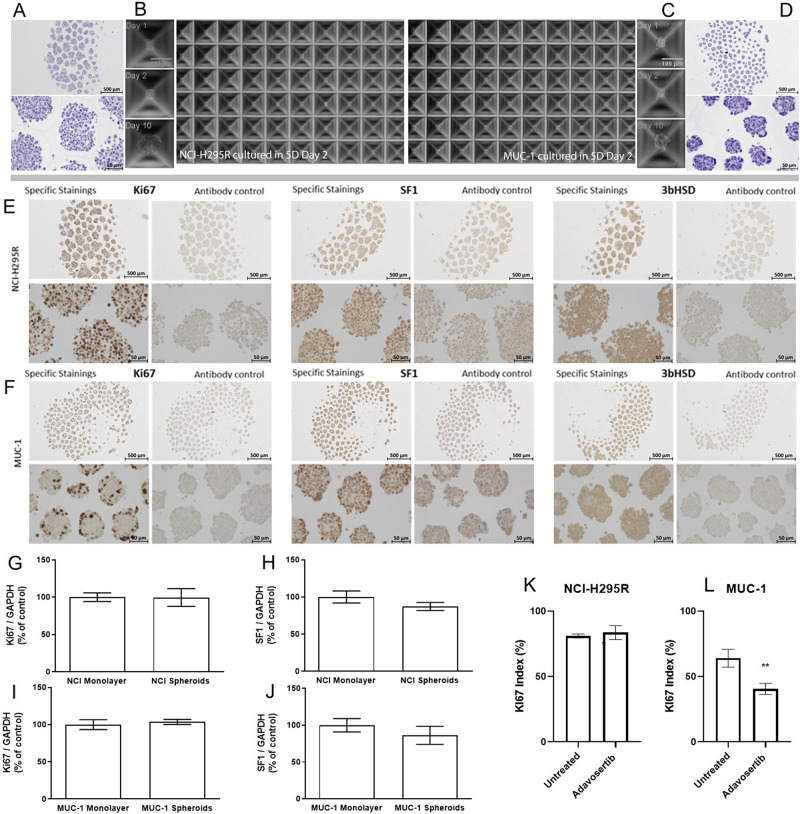
NCI-H295R and MUC-1 cell lines form spheroids in the Sphericalplate 5D. H&E staining of NCI-H295R spheroids **A** and MUC-1 spheroids **D** and progression of spheroid formation **B** and **C** respectively) over 10 days. NCI-H295R spheroids **E** and MUC-1 spheroids **F** were processed for IHC with antibodies against Ki67, SF-1 and 3bHSD, scale bar 500 µm and 50 µm. Gene expression of Ki-67 **G** and SF-1 **H** was compared by real-time RT-PCR in NCI-H295R monolayer cells and spheroids (*n* = 3). Gene expression of Ki67 **I** and SF-1 **J** was compared by real-time RT-PCR in MUC-1 monolayer cells and spheroids. Ki67 index between control and Adavosertib-treated NCI-H295R **K** and MUC-1 **L** spheroids. Data analyzed by Student’s t-test, **p < 0.01.

Next, for translation to adrenal-related applications, the human adrenocortical cell lines NCI-H295R and MUC-1 were seeded to Sphericalplates and investigated for successful tumor spheroid formation as well as ongoing spheroid growth (Fig. 3B, C). Histological and immunohistochemical stainings confirmed viable spheroids (Fig. 3A, D), which reflected tumor model specific proliferation rates (Ki67) and steroidogenic activities as demonstrated by SF-1 and 3betaHSD positivity (Fig. 3E, F).

Subsequent real time PCR investigations for potential changes in Ki-67 or SF-1 gene expression levels in tumor spheroid versus the appropriate monolayer cultures, did not reveal significant differences (Fig. 3G–J). Pilot experiments investigating the calculation of Ki-67 Index upon specific drug treatments as important pathological determinant, demonstrated furthermore applicability of such readouts. In a therapeutic setting, the quantification of Ki-67 indices revealed significant anti-proliferative effects of adavosertib, a small molecular Wee1-inhibitor, against MUC-1 but not NCI-H295R tumor spheroids (Fig. 3K, L). These results were in agreement with parallel MTT experiments on monolayer cultures (data not shown).

### Establishment and characterization of patient-derived tumor spheroids from adrenocortical origin

**Fig. 4:**
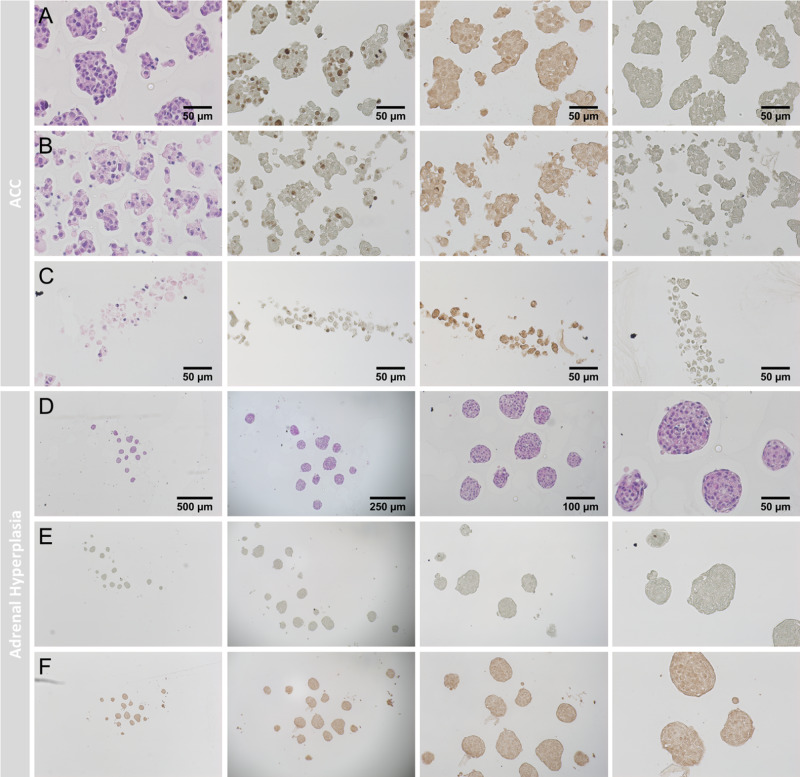
Spheroid formation from patient-derived ACC investigated by H&E, Ki-67, SF-1 and 3bHSD (from left to right). Control spheroids **A**, spheroids treated with 25 µM Gemcitabine **B** and spheroids treated with Gemcitabine (25 µM) and Cisplatin (40 µM, **C**). Patient-derived spheroids from benign adrenal hyperplasia investigated by H&E **D**, Ki-67 **E** and SF-1 **F**.

To extend the established adrenal tumor models for applications in terms of personalized medicine, we aimed furthermore at the development of patient-derived tumor spheroids. Fig. 4A demonstrates appropriate H&E, Ki-67, SF-1 and 3betaHSD stainings which overall accounted for viable and proliferating spheroids derived from a primary ACC.

Even though elevated background staining was detectable for these spheroids, the experiments indicated clearly definable nuclear SF-1 positivity compared to the appropriate antibody controls, but rather low or absent 3betaHSD abundance. In a therapeutic setting, histologically weak anti-tumoral effects were already detectable upon treatment with 25 µM Gemcitabine (Fig. 4B). In contrast, Fig. 4C demonstrates severe pathological changes upon treatment with 25 µM Gemcitabine and 40 µM Cisplatin.

Interestingly, not only malignant tumors can be cultured by our method, Fig. 4D shows viable spheroids of a benign adrenal hyperplasia, with the characteristic low/lacking Ki-67-Index (Fig. 4E) compared to ACCs (Fig. 4A). These tumor-spheroids were furthermore SF-1 positive (Fig. 4F), but 3betaHSD negative (data not shown).

### Establishment and characterization of patient-derived tumor spheroids from medullary origin

**Fig. 5:**
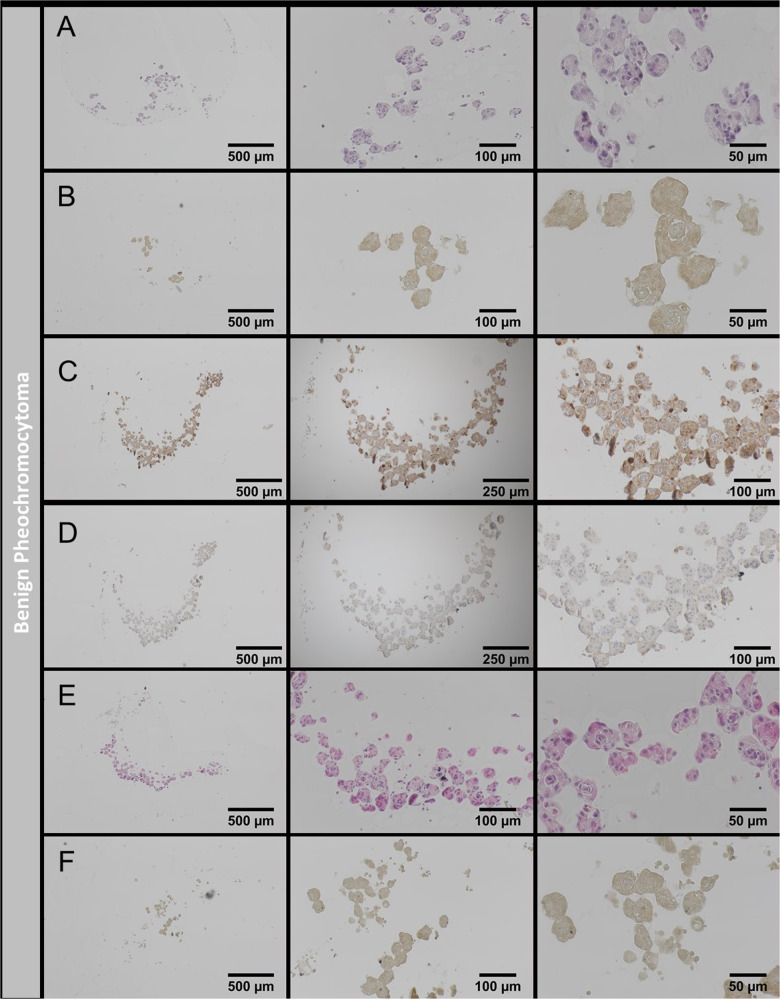
Spheroid formation from benign pheochromocytoma. H&E staining of spheroids at 3 different magnifications **A**. Spheroids were analyzed with the proliferation marker Ki-67 **B**. Spheroids processed for chromogranin A **C** and its respective no-antibody control **D**. Spheroids treated with 25 µm Gemcitabine (H&E, **E** and Ki-67, **F**).

Despite the need of innovative multi-dimensional and patient-derived in vitro models for adrenocortical tumors, there is nowadays also still a fundamental lack of models of medullary origin with no human cell line available. In an attempt to investigate putative applicability also for this tumor entity, we cultured cells freshly obtained from a surgical tumor sample of a benign pheochromocytoma. Of note, as demonstrated in Fig. 5, we obtained viable tumor-spheroids (5A), which showed no proliferation in vitro (5B), but high functionality as indicated by strong chromogranin A stainings (Fig. 5C vs. appropriate antibody controls 5D). Test-wise therapeutic administration of 25 µM Gemcitabine, confirmed again applicability of such read-outs. However, patient-individual the treatment resulted for these spheroids in no or only mild therapeutic response if compared with the pictures above for ACC tumor spheroids.

### Establishment and characterization of organoids from normal adrenal origin (bovine and porcine)

**Fig. 6:**
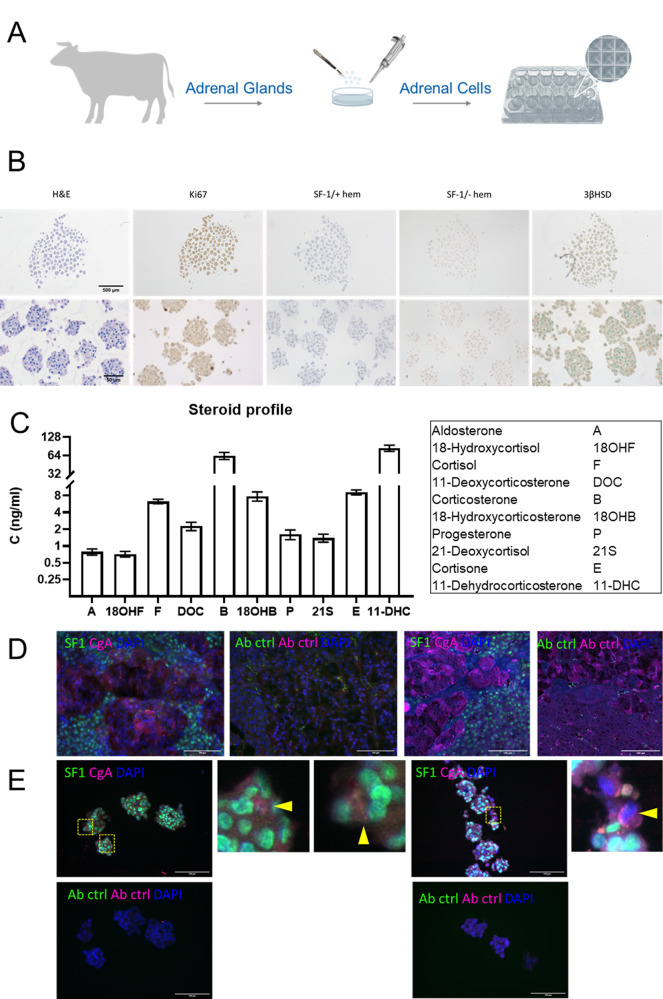
Successful formation of organoids from bovine cells of adrenocortical and medullary origin. Scheme depicting experimental set up **A**. Organoids processed for H&E, Ki67, SF-1 (with and without hematoxylin counterstain) and 3bHSD staining at two different magnifications **B**. Secretory profile of organoids derived from a bovine adrenal gland as analyzed by LC-MS/MS (*n* = 3) **C**. Bovine adrenal sections stained with antibodies against SF-1 and chromogranin A (CgA), scale 100 µm **D**. Adrenocortical and medullary cells in organoids labeled with SF-1 and chromogranin A, scale 100 µm **E**.

Next, we aimed at the development of multi-dimensional models obtained from healthy adrenal glands (Fig. 6).

**Fig. 7:**
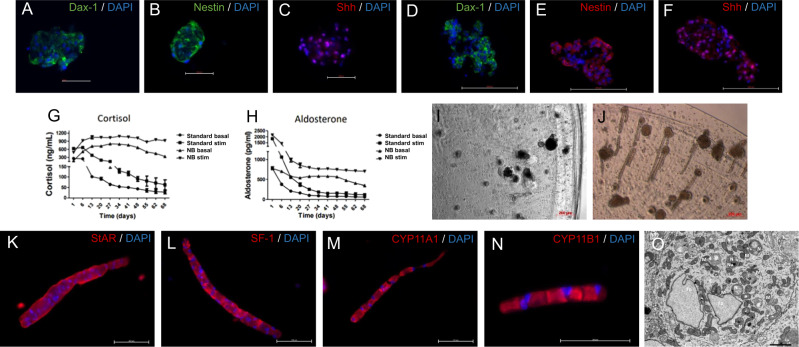
Investigation of healthy bovine and porcine derived adrenal 3D-cultures. Spheroids from bovine (**A**–**C**) and porcine (**D**–**F**) adrenals stained with antibodies against DAX1, Nestin and SHH. Effect of growth media on the secretion of cortisol **G** or aldosterone **H** into the supernatant of porcine adrenal spheroids (3 experiments with 4 replicates each). Images of porcine spheroids **I** or spheroids treated with ATCH **J** highlight the presence of multicellular extensions following ACTH stimulation. Multicellular extensions expressed the markers StAR, SF-1 CYP11A1 and CYP11B1 (**K**–**N**). Electron micrograph of adrenocortical cells, migrating from the spheroid **O**. The cell shows characteristic features of mature steroid producing cell including characteristic elongated and round- shape mitochondria (mit) with the typical tubulovesicular internal membranes. Furthermore, cells exhibit ample smooth endoplasmic reticulum (SER) and cell membrane extensions in form of microvilli (mv), scale 2 µm.

For this purpose, we made cross-sections from healthy adrenals to reflect this time cells of both, adrenocortical and medullary origin, in vitro in one well (Fig. 6A). Bovine cells were subsequently cultured in Sphericalplates and as summarized in Fig. 6B our experiments revealed viable and as for the healthy tissue origin expected low/not proliferating organoids. SF-1/3betaHSD immunohistochemistries and LC-MS/MS based adrenocortical steroid profiling confirmed specific basal steroidogenic activity (Fig. 6B, C). Subsequent SF-1 and CgA immunefluorescence co-stainings verified furthermore viable co-cultures of cells specifically derived from both, adrenocortical and medullary origin (Fig. 6D bovine adrenal tissue controls, 6E different organoids including antibody controls) indicating the potential of these models to mimic dynamic zonation processes or zone to zone communication in vitro. In parallel, we currently investigate spheroids of bovine (Fig. 7A–C) vs. porcine (Fig. 7D–F) origin for potentially best applicability in terms of clinical transplantations for the treatment of diseases such as adrenal insufficiency. Both types demonstrate abundance of relevant markers such as DAX-1, Nestin and Sonic hedgehog indicating, thereby, stem cell and adrenal regenerative properties. Interestingly, culturing of porcine adrenal spheroids in NB-Medium led furthermore to highly improved cortisol and aldosterone output under basal and ACTH-stimulated conditions (Fig. 7G, H). Moreover, multicellular extension-formation could be observed under ACTH-treatment exclusively (Fig. 7J) compared to controls (Fig. 7I). Of note, these types of multicellular filopodia demonstrated adrenal specific StAR, SF-1, CYP11A1 and CYP11B1 protein abundances (Fig. 7K–N). Electron microscopy showed furthermore, that these structures indeed originate from zona fasciculata cells (Fig. 7O).

## Discussion

Here we report on the development of a multi-well plate created for multi-dimensional cell modelling and its applicability for different endocrine cell and tissue sources. As culturing of small pseudo-islet has been previously demonstrated to be advantegous over large pancreatic islets [24] and is since then commonly applied in the field of diabetology, we demonstrate in our study for pseudo-islets only the general applicability for such approaches. However, additionally indicated benefits, such as round tip wells and no need for otherwise commonly applied anti-adherent buffers, can be also taken as further advantages for pseudo-islet cultivation into account.

However, the focus of this study was on multidimensional models derived from various adrenal tissue origins (from healthy over benign to malignant and adrenocortical to medullary cells) as within the adrenal field such approaches are yet not widely established. Potential implementations range from basic research, cancer research and furthermore include organ replacement therapies for adrenal insufficiency [5, 6]. Moreover, chromaffin cells from the adrenal medulla have been also considered e.g. as a potential source of dopamine-producing cells to treat neurodegenerative conditions like Parkinson disease or pain [7]. Even though first attempts in this direction have been recently published and organoids demonstrated to retain viability and expression of zonal and functional markers, this approach was limited to human fetal adrenal cell population and did not provide developmental potential for or implementation of post-natal tissues. Moreover, this technique and up to our knowledge all others applied until today in the adrenak field, clearly lack high-throughput applicability [25].

In addition, our group previously demonstrated that alginate-encapsulated bovine adrenal spheroids can be a cell source for xenogeneic transplantation for the treatment of adrenocortical deficiency [26]. However, while originally developed to keep the cells isolated from the host immune response by a semipermeable membrane, it is known now that the immune system recognizes pathogen associated molecular patterns in alginate preparations themselves, thereby leading to adaptive and innate immune responses against engrafted capsules. Accordingly, the few clinical trials based on such alginate involving bioengineering approaches did finally not lead to any licensed therapy [27]. In addition, there is substantial heterogeneity of various alginate capsules regarding the maximum permeability ranging from 25 to 250 kDa. These are additional factors which can also impact the normal exchange of spheroids regarding e.g. proteins and polysaccharides with their natural environment and thereby also viability [27]. In contrast, the innovative spherical plate implemented in our novel approach, enables spheroid growth on a medical grade ultra-low attachment coating without any need for encapsulation and even without additional need for otherwise commonly required anti-adherence rinsing solutions that can potentially interfere with cell biology or hamper medical use. Moreover, the spherical plate enables a barrier-free exchange of spheroids and organoids with their appropriate environment and our data confirm viability as well as specific hormonal functionality. Of note, recently it has been shown that co-culturing of mouse or porcine cells together with CXCL12 leads to immunoprotection and long-term function with no further need of systemic host immunosuppression [28]. Consequently, we currently aim in parallel at investigations on porcine adrenal cells, their extra-ordinary regenerative potential and thereby optimized suitability for such approaches without or with low immune-activating stabilization in the future patient. Our most recent experiments in this direction demonstrate, that while basal DAX1, Nestin and SHH abundances between bovine and porcine cells seemed to be comparable, the newly discovered ACTH-dependent potency to differentiate to StAR, SF-1, CYP11A1 and CYP11B1 consisting multicellular extensions indicate the extraordinary regenerative potential of porcine cell populations in this context. Accordingly, in next steps we aim to combine and optimize our findings specifically for the establishment of high-throughput porcine derived adrenal spheroids immune-optimized for transplantation purposes.

Overall, our report demonstrates the most innovative and complete approach for 3D-modelling starting from highly varying adrenal tissue sources. Up to date, except limited description of classically cultured NCI-H295R spheroids [29–31], to our knowledge no comparable report, practical approach and appropriate protocol has been reported so far for adrenal tumors. Moreover, our provided protocols allow also high-throughput-applicability in terms of drug testings including subsequent histological/immunohistochemical readouts (such as classical Ki-67 indices or specific stainings regarding endocrine functionality) and thereby highly comparable applicability for personalized medical approaches in appropriate endocrine tumor boards.

## Materials and methods

### Cell culture

#### Human pancreatic islets

Human islets were obtained by the Islets for Research Distribution Programme of the European Consortium for Islet Transplantation (ECIT; biological replicates *n* = 10, technical replicates *n* = 3 from three high power fields per individual). The providing centres were the Cell Isolation and Transplantation Centre, Geneva University Hospitals; the Islet Processing Facility, S. Raffaele Scientific Institute Milan; “Recherche Translationnelle sur le Diabète” Faculté de Médecine, Université de Lille/CHRU de Lille/Inserm/EGID; and from Prodo Laboratories, Aliso Viejo CA. They were cultivated, dissociated into single cells and reaggregated as described previously [32]. Dissociated cells were reaggregated in Sphericalplates 5D (Kugelmeiers, Erlenbach, Switzerland) at the required concentration in 0.5 ml medium (e.g. 300 cells/microwell, for 750 microwells/well, 225,000 cells/0.5 ml). Medium was changed every second day. Islet perifusion was performed as described [32] except the device used was the Perifusion System (Model No: Peri-4.2, Biorep Technologies, Miami, FL).

#### Adrenocortical cell lines, primary culture and tumor spheroids

Primary cultures of patient tumors were established as previously reported [13]. Tumor sample collection was approved by the local ethics committee (Kantonale Ethikkommission Zürich, BASEC 2017-00771) and written informed consent was obtained from all subjects prior to tumor sampling. Cells from benign pheochromocytoma and benign adrenal hyperplasia were plated at 250,000 cells /well in the spherical plate 5D (Kugelmeiers, Erlenbach, Switzerland). NCI-H295R, MUC-1 and ACC115m primary cells were cultured as previously described [13, 18] and cultivated in Sphericalplates 5D for either 7 or 14 days at the densities of 75,000 cells/well, 150,000 cells/well and 75,000 cells/well respectively. The 7 day spheroids were treated with 650 nM Adavosertib (MedChemExpress) for 72 h. Additionally, ACC115m cells were cultivated in Sphericalplates 5D for 7 days and subsequently treated with 25 µM Gemcitabine and 40 µM Cisplatin (both Teva Pharma) for 72 h.

#### Adrenal spheroids

For 5D-cultured adrenal organoids and subsequent LC-MS/MS measurement of appropriate supernatants a 1.22 g cross-section piece of bovine adrenal gland was minced to less than 0.5 mm pieces using a razor blade in PBS. The resulting suspension was centrifuged at 250 g for 5 min and the pellet was incubated with 2 mg/ml sterile filtered Collagenase II (Gibco, Waltham) in DPBS for 50 min at 37 °C. Collagenase was inactivated with 5 ml FBS (Thermo Fisher Scientific). Cells were pelleted and erythrocyte lysis was performed with 5 ml RBC lysis buffer (pH 7.4) for 7 min at room temperature (150 mM ammonium chloride, 1 mM potassium hydrogen carbonate and 0.1 mM disodium-EDTA). Upon another centrifugation step, cell pellet was resuspended in PBS, and, after straining through a 70 µm mesh, cells were counted using a Neubauer Improved hemocytometer, and centrifuged again at 250 g for 5 min. Dead cells were removed using Dead Cell Removal Kit (Miltenyi Biotec). Live cells were resuspended in Advanced DMEM/F-12 medium containing 17.51 mM D-glucose, non-essential amino acids, and 1 mM sodium pyruvate supplemented with 10% FBS and 1% penicillin/streptomycin (all Thermo Fisher Scientific), and seeded in Sphericalplates 5D at a density of 250,000 cells/well.

For the investigation of stem cell and differentiation characteristics bovine adrenocortical cells were isolated from bovine adrenal glands shortly after the slaughtering of 1–3 years old cattle as previously described [26]. Briefly, adrenal glands were transported to the laboratory in ice-cold Euro Collins Solution supplemented with 1% (vol/vol) penicillin-streptomycin solution (Thermo Fisher Scientific). The glands were then liberated from fat and connective tissue and rinsed several times with PBS through the central vein to remove remaining blood. Afterwards a longitudinal incision was made to cut the adrenals in halves, the medulla was removed and the cortex was scraped off the capsule and cut in small pieces. Adrenal capsule was discarded. Adrenal cortex was digested for 50 min in DMEM/F12 medium (Thermo Fisher Scientific), containing 2 mg/ml collagenase and 0.1 mg/ml DNase (both from Sigma-Aldrich) at 37 °C while shaking. Obtained cells were washed with cultivation medium, pelleted by centrifugation (8 min, 300 g) and filtered through 100-µm cell strainers (Becton Dickinson). After that, primary adrenocortical cells were placed in cell culture flasks (Thermo Fisher Scientific) and cultivated at 37 °C in a humidified atmosphere (95% air, 5% CO_2_) in DMEM/F12 medium with 10% (vol/vol) FBS, 10% (vol/vol) horse serum (both from Thermo Fisher Scientific), 0.1 ng/ml recombinant FGF-2 (PromoCell GmbH) and 1% (vol/vol) penicillin-streptomycin solution. Medium change was performed every 2–3 days. Cells remained in culture for 4 days.

For spheroid formation, frozen cells were thawed, resuspended in standard medium and cultivated for 3 days. Cells were then trypsinized and placed in cell culture inserts (1 × 10^6^ cells per insert) with PTFE membrane (Merck) in 6 well plates for overnight cultivation. The next day the spheroids from each insert were mixed gently with 150 µl of 2% alginate (previsously described [26], placed 30 µl of the mixture on a glass with 550 micron spacers, covered with flat Sintered glass (Pyrex) and cross-linked with 70 mM strontium chloride for 10 min. Received slabs were cultivated in neurobasal medium (Neurobasal medium, containing 2% (vol/vol) of B-27 supplement, 2 mM L-glutamine (all from Thermo Fisher Scientific), 20 ng/ml FGF -2 (Promocell) and 1% (vol/vol) penicillin-streptomycin-nystatin).

#### Porcine adrenal spheroids

Porcine adrenal cells were isolated from adrenals of freshly slaughtered pigs. During the transportation and cell isolation, all solutions, except enzyme, contained 1% (vol/vol) of penicillin-streptomycin-nystatin (Neolab). The glands were transported to the laboratory in cold Euro-Collins solution, freed of connective and adipose tissue and washed twice in PBS. Then the glands were cut in half by a longitudinal incision, the cortex with medulla were scrapped off the capsule, cut into small pieces, and digested for 50 min in DMEM/F12 (Gibco, Thermo Fisher Scientific), containing 2 mg/ml collagenase type V (Sigma–Aldrich), and 0.1 mg/ml DNase (Roche) at 37 °C while shaking. The cells were then washed twice in standard cultivation medium and pelleted by centrifugation (8 min, 300 × *g*) and filtered through gauze. Primary adrenal cells were cultivated in standard medium (DMEM/F12 with 5% (vol/vol) FBS, 10% (vol/vol) horse serum (both from Gibco, Thermo Fisher Scientific), 10 µg/ml insulin (Sanofi), 5 µg/ml transferrin (Sigma–Aldrich) and 1% penicillin-streptomycin-nystatin) at 37 °C in a humidified atmosphere (95% air, 5% CO_2_). On the day following cell isolation, the cells were removed from the cell culture by trypsinization (TrypLE™, Thermo Fisher Scientific), pelleted by centrifugation, resuspeneded in a freeze medium (standard medium, containing 10% (vol/vol) DMSO) and stored frozen in the liquid nitrogen.

### Immunohistochemistry

#### Pancreatic pseudo-islets

Spheroids were fixed with 4% paraformaldehyde (PFA, Sigma–Aldrich), embedded in Histogel (Thermo Fisher Scientific), then dehydrated and processed for paraffin embedding using the Excelsior AS tissue processor (Thermo Fisher Scientific). Spheroids were embedded using Myr embedding center (Especialidades Médicas MYR) and 4 µm sections were prepared (microtome Leica RM2255, Leica Biosystems). For Ki67 staining, slides were processed with the Autostainer Link48 (DAKO, Agilent) and all reagents were purchased from DAKO. For all other antibodies, sections were deparaffinized, rehydrated, and, following the HIER treatment in sodium citrate buffer, incubated with blocking buffer containing 3% BSA (Roche Diagnostics), 5% goat serum (Jackson ImmunoResearch Laboratories), and 0.5% Tween 20 (Sigma–Aldrich). Sections were incubated with the following primary antibodies overnight: Ki67 (1:100, IR626, DAKO), cleaved caspase 3 (1:300, 9661, Cell Signaling), insulin (1:200, I2018Sigma–Aldrich), glucagon (1:50, PA5-13442, ThermoFisher), ppy (1:200, RP030-05, Diagnostic BioSystems) and somatostatin (1:250, A0566, DAKO). The following day, sectiones were either processed with the Vectastain Elte ABC-HRP kit (Vector Laboratories) and visualized by DAB (Sigma–Aldrich) or detected with fluorescent secondary antibodies (Jackson ImmunoResearch). Images were processed with Zeiss Axiovert 200 M with AxioCamMRc5 (6 images/slide).

#### Adrenal spheroids

Spheroids were processed as outlined in the pancreatic islet section. Primary antibodies used with the adrenal cortical cell lines were incubated overnight at 4 °C and were as follows: Ki67 (1:200, DCS), SF-1 (1:200, Cosmo Bio) and 3β-HSD1 (1:1500, provided by Anita Payne, Stanford University School of Medicine, Stanford, CA, USA). Secondary antibody (1:200 goat anti-rabbit biotinylated IgG, Vector Laboratories) was applied at room temperature for 1 h for Ki67 and 3β-HSD1 resp. for 40 min for SF-1. Following the incubation with the ABC solution (Vectastain Elite ABC-HRP Kit, Vector Laboratories), bound antibodies were visualized by 3,3´-diaminobenzidine staining (for Ki67 and 3β-HSD1: Sigma–Aldrich, for SF-1: Vector Laboratories).

Immunofluorescence stainings and imaging of spheroid sections were performed as described previously [33] using following antibodies: SF-1 (1:200, Cosmo Bio), Chromagranin A (1:100, Santa Cruz Biotechnology), donkey anti-goat-Cy3 (1:500, Jackson ImmunoResearch), and donkey anti-rabbit Alexa Fluor 647 (1:500, Invitrogen).

### Immunofluorescence of bovine and porcine adrenal spheroids

Free floating adrenal spheroids were fixed overnight in 4% PFA and embedded in Tissue-Tek O.C.T (Sakura Finetek). Immunohistochemistry was performed on 6-μm sections using primary antibodies for DAX1 (1:100, LS-B3480, LSbio), Nestin (1:100, NBP1-02419, Novusbio), SHH (1:100, LS-C40460, LSbio), Following overnight incubation with primary antibodies, sections were washed and incubated with for 1 h at room temperature with secondary antibodies goat anti-rabbit Alexa Flour 568 (1:1000, A11011; Life Technologies) for Nestin, SHH, or goat anti-rabbit Alexa Flour 488 (1:1000, A11034, Life Technologies) for DAX1, nestin or goat anti-mouse Alexa Flour 488 (1:1000, A11001, Life Technologies). DAPI (10236276001; Roche) was used for cell nucleus-specific staining. Immunofluorescence microscopy was performed on an Axioplan Carl Zeiss microscope, using Axiovision Software, or on Zeiss Axiovert 200 M with AxioCamMRc5, using ZEN 3.3 (Carl Zeiss) software.

Spheroids encapsulated in alginate and adrenal column were washed with PBS and fixed with 4% PFA overnight. Since the fixative is hypo-osmotic, during the fixation procedure the alginate swells, which increases its porosity. After washing with PBS, the slabs were incubated in blocking solution (3% BSA, 0.1% Triton X-100 in PBS for 30 min at room temperature), then incubated with the primary antibodies DAX1, SHH, WNT4 (1:100, ab91226, Abcam), StAR (1:100, sc-25806, Santa Cruz Biotechnology), SF1, CYP11A1 (1:100, sc-18043, Santa Cruz Biotechnology), and CYP11B1 overnight at 4 °C. The following day, slabs were washed with PBS and incubated for 1 h with the secondary antibody (goat anti-rabbit Cy3-conjugated IgG, 1:500, 111-65-144, Jackson Immunoresearch laboratories). Cell nuclei were stained with DAPI. Leftovers of alginate were removed by incubating in 500 mg/ml (w/v) solution of alginate lyase (Sigma–Aldrich) for 30 min. Immunofluorescence microscopy was performed on Axioplan microscope, using Axiovision Software.

### Transmission electron microscopy (TEM)

The spheroids were fixed in 4% PFA in 0.1 M phosphate buffer (PB, pH 7.4), followed by postfixation in modified Karnovsky’s fixative (2% glutaraldehyde +2% paraformaldehyde in 50 mM HEPES, pH 7.4) overnight at 4 °C [34, 35]. Samples were washed 2x in 100 mM PB and 2x in water and postfixed in 2% aqueous OsO4 solution containing 1.5% potassium ferrocyanide and 2 mM CaCl2 for 30 min on ice. After washes in water, the samples were incubated in 1% thiocarbohydrazide in water (20 min at room temperature), followed by washes in water and a second osmium contrasting step in 2% OsO4/water (30 min, on ice). Samples were washed in water, en bloc contrasted with 1% uranyl acetate/water for 2 h on ice, washed again in water, dehydrated in a graded series of ethanol/water (30%, 50%, 70%, 90%, 96%, 3 × 100% ethanol (pure ethanol on molecular sieve)), and infiltrated in the epon substitute EMBed 812 (epon/ethanol mixtures: 1:3, 1:1, 3:1 for 1.5 h each, pure resin overnight, pure resin 5 h). Finally, the samples were embedded in flat embedding molds and cured at 65 °C overnight. Ultrathin sections were prepared with a Leica UC6 ultramicrotome (Leica Microsystems, Vienna, Austria), collected on formvar-coated slot grids, and stained with lead citrate (Venable and Coggeshall, 1965) and uranyl acetate. Contrasted ultrathin sections were analysed on a Jeol JEM1400 Plus (JEOL, Freising, Germany, camera: Ruby, JEOL) running with 80 kV acceleration voltage.

### Steroid and insulin measurements (LC-MS/MS and ELISA)

For functional assessment of steroid hormone release by adrenal spheroids liquid chromatography tandem mass spectrometry (LC-MS/MS, technical replicates *n* = 3) was applied as described elsewhere [36].

For basal and ACTH stimulated steroid release, the slabs were incubated with or without 3 ng/ml ACTH_1-24_ (Synachthen, Sigma-tau Arzneimittel GmbH) for 24 h. Measurements of cortisol, aldosterone or insulin in cell culture supernatants were done by cortisol, aldosterone (IBL) and insulin (mercodia Cat. Nr. 10-1113-01) ELISA, respectively.

### Real-time PCR

Spheroid and monolayer samples (technical replicates *n* = 3) of MUC-1 and NCI-H295R cells were processed for RNA extraction using the RNeasy Mini kit (Qiagen), followed by DNA removal (TURBO DNA-free™ Kit, Thermo Fisher Scientific) and reverse transcription (RevertAid™ H Minus First Strand cDNA Synthesis Kit, Thermo Fisher Scientific) where 310 ng of RNA was converted to cDNA per sample. For Real-Time PCR analyses, we utilized the SsoFast EvaGreen^®^ reaction mix (Bio-Rad Laboratories) in the 7500fast cycler (Applied Biosystems). The following PCR primers were used: human SF1 (forward: 5′- CAGCCTGGATTTGAAGTTCCT, reverse: 5′- CAGCATTTCGATGAGCAGGT) and human Ki67 (forward: 5′- TCCTTTGGTGGGCACCTAAGACCTG, reverse: 5′- TGATGGTTGAGGCTGTTCCTTGATG). Gene expression levels were normalized to the housekeeping gene GAPDH (forward: 5′-AGCCTCCCGCTTCGCTCTCT, reverse: 5′-CCAGGCGCCCAATACGACCA).

### Statistical analysis

Statistical analysis and graphical representation of the data was carried out using GraphPad Prism software (version 8, GraphPad Software, La Jolla, CA, USA). Statistical comparisons were performed using one-way ANOVA followed by Bonferroni or Dunnett’s multiple comparisons test. All results are expressed as mean ± SEM. The statistical significance was defined as *P* < 0.05 and denoted as stars in the graphs (*p < 0.05; **p < 0.01; ***p < 0.001; ****p < 0.0001) in all figures, if not stated otherwise.

## Supplementary information

Checklist

## Data Availability

All data that were needed to evaluate the conclusions in the paper are present in the paper. The cell line MUC-1 can be provided by C. Hantel pending scientific review and completed MTAs.
